# Microstructure and Properties of Nickel-Based Gradient Coatings Prepared Using Cold Spraying Combined with Laser Cladding Methods

**DOI:** 10.3390/ma16041627

**Published:** 2023-02-15

**Authors:** Sainan Liu, Yangyang Sun, Pengyuan Zhai, Pengyu Fan, Yongtong Zhang, Muyang Li, Jianxiao Fang, Ruilin Wu, Zhenyang Cai

**Affiliations:** 1 School of Minerals Processing and Bioengineering, Central South University, Changsha 410083, China; 2 New Technology Promotion Institute of China Ordnance Industries, Beijing 100089, China; 3 Henan Jianghe Machinery Co., Ltd., Pingdingshan 467000, China; 4 School of Materials Science and Engineering, Central South University, Changsha 410083, China

**Keywords:** cold spraying, laser cladding, nickel-based gradient coating, wear properties

## Abstract

A cold spray–laser cladding composite gradient coating (CLGC) was successfully formed on a Cu substrate. In comparison with traditional laser cladding gradient coatings (LGC), cold spraying the pre-set Ni-Cu alloy’s intermediate transition layer not only mitigates the negative impacts due to the high reflectivity of the copper substrate but also helps to minimize the difference in the coefficients of thermal expansion (CTE) between the substrate and coating. This reduces the overall crack sensitivity and improves the cladding quality of the coating. Besides this, the uniform distribution of hard phases in CLGC, such as Ni_11_Si_12_ and Mo_5_Si_3_, greatly increases its microhardness compared to the Cu substrate, thus resulting in the value of 478.8 HV_0.5_ being approximately 8 times that of the Cu substrate. The friction coefficient of CLGC is lowered compared to both the Cu substrate and LGC with respective values of 0.28, 0.54, and 0.43, and its wear rate is only one-third of the Cu substrate’s. These results suggest CLGC has excellent anti-wear properties. In addition, the wear mechanism was determined from the microscopic morphology and element distribution and was found to be oxidative and abrasive. This approach combines cold spraying and laser cladding to form a nickel-based gradient coating on a Cu substrate without cracks, holes, or other faults, thus improving the wear resistance of the Cu substrate and improving its usability.

## 1. Introduction

Favorable properties, including good electrical and thermal conductivity, ease of processing, etc., have resulted in copper and its alloys being essential in the fields of rail transportation, mechanical engineering, and aerospace [1,2]. However, the strength and wear resistance requirements of certain fields cannot be met by traditional copper alloys [3,4,5]. As an advanced surface modification technology, laser cladding (LC) forms high-performance coating with metallurgical bonding on Cu substrates by using a high-energy laser beam and thus improving the surface strength and wear resistance of the Cu substrate greatly [6,7,8,9]. Nevertheless, it is difficult to laser clad the surfaces of copper alloys with a crack-free, metallurgically bonded, wear-resistant cladding layer due to their low wettability with a variety of materials and the high reflectivity of the laser [10,11].

The introduction of nickel-based intermediate layers using pre-set laser cladding is regarded as a viable solution [12,13]. On the one hand, this effectively improves the reflectivity of the substrate to the laser, increases the powder utilization rate, and lowers the cost due to the pre-placement of the powder on the surface of the Cu substrate. On the other hand, adding the appropriate amount of nickel elements can reduce the thermal conductivity of Cu substrates and promote the formation of a melt pool during the laser cladding process as a consequence of the infinite solid solution of nickel and copper in the phase diagram [14,15,16]. However, increasing the internal porosity, surface contamination, or severe oxidation during the cladding process represent the common problems associated with the introduction of intermediate layers using pre-setting methods such as bonding or thermal spraying [17,18,19,20,21,22,23]. The high coating density, low oxidation, and low thermal impact on the substrate are the advantages of using cold spraying (CS), which is a new surface coating technology, compared with the traditional plasma spraying and flame spraying [24,25,26]. These advantages result in the efficient preposition of the nickel-based intermediate layer without affecting the organization and performance of the Cu substrate. Although cold spraying or laser cladding has been used to prepare nickel-based coatings on copper-based surfaces, the combination of the cold spraying pre-deposition with laser cladding has not been used for the preparation of nickel-based gradient wear-resistant coatings [14,27,28,29,30,31].

Herein, cold spraying was innovatively used to pre-deposit the Ni-Cu alloy coating on the Cu substrate surface. This formed a Ni-Cu-alloyed layer by using laser alloying process and was followed by a high-speed laser cladding process to fuse nickel-based powder on the Ni-Cu-alloyed layer surface to finally obtain a crack-free, wear-resistant, composition and performance gradient nickel-based wear-resistant coating. The coating significantly improved the surface strength and wear resistance of the Cu substrate.

## 2. Materials and Methods

### 2.1. Feedstock Powder and Substrate

The chemical composition of the powder feedstock and the Cu substrate with a size of 60 mm × 60 mm × 8 mm is shown in Table 1. The table shows that the Ni content decreases from the first to the fourth layer, while the trend of the other elements (Mo, W, and Si) reverses, resulting in a uniform transition between the composition of each layer. The first layer’s function is to produce a metallurgical bonding layer with the copper substrate for strengthening the bonding between the gradient coating and copper substrate as well as reducing the difference in the coefficients of thermal expansion (CTE) between them (1.1 × 10^−5^ k^−1^ for the first layer and 1.94 × 10^−5^ k^−1^ for the Cu substrate) [32]. On the other hand, the second to fourth layers are supposed to improve its wear resistance because of the gradually increasing hardness of the gradient coating. At the same time, ZrO_2_-5.4%Y_2_O_3_(YSZ) with a particle size of 100 nm was used in this experiment to help refine the grain size and enhance the wear resistance of the coating. In addition, the same process parameters were used to prepare the laser cladding gradient coating (LGC) without a cold spray pre-set first layer and were used as comparative samples. All the experiments and performance testing were carried out employing the same equipment.

**Figure 1:**
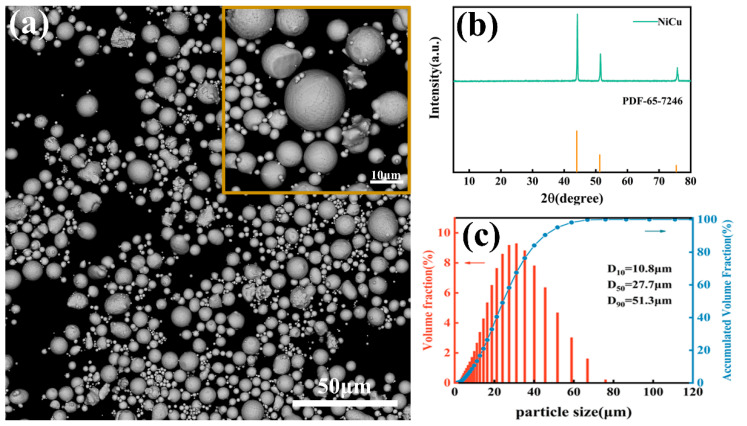
(**a**) SEM and (**b**) XRD analysis, (**c**) particle size distribution of the cold spray powder.

In particular, the SEM and XRD analysis of the powder used for the base layer (first layer) to prepare the Ni-Cu alloy powder using high pressure cold spraying is shown in Figure 1a,b, respectively, and its particle size distribution, measured using a Mastersizer 3000 laser diffraction particle analyzer (Malvern Instruments Ltd., Malvern, UK), can be seen in Figure 1c (D_10_ = 10.8 μm, D_50_ = 27.7 μm and D_90_ = 51.3 μm).

### 2.2. Preparation of Gradient Coating

**Figure 2:**
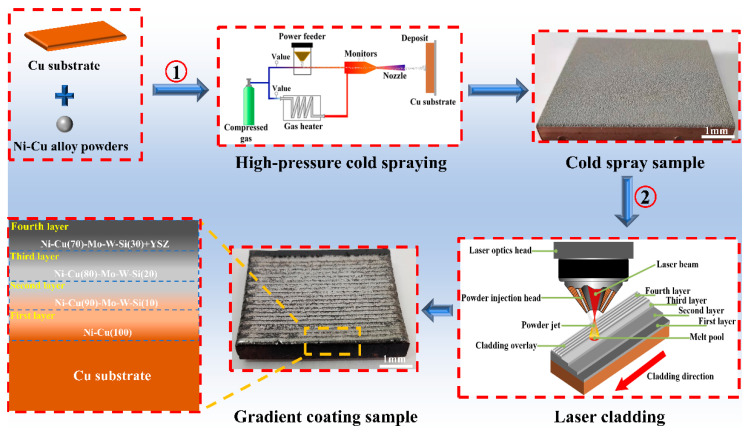
Schematic diagram of the gradient coating preparation process.

The preparation process of a cold spray–laser cladding composite gradient coating (CLGC) is shown in Figure 2. A Psc-100 cold spraying system (Nippon Plasma Giken Kogyo Co., Saitama, Japan) was used to prepare the first layer. To prepare the pure Cu substrate for cold spraying, the surface of the pure Cu substrate was sandblasted for two purposes: first, cleaning of the surface as well as roughening it and, second, enabling a stronger bond between the coating and substrate. After that, the substrate was cleaned under ultrasound for 10 min and then dried. High-purity nitrogen was used both as an accelerator and carrier gas during the cold spraying process. The optimum cold spraying process was determined based on the porosity and surface roughness values. Subsequently, the obtained samples based on the optimal cold spraying process were preheated in an oven at 200 °C for 0.5 h and then underwent the laser cladding process using ZKZM-10000 W high speed laser cladding equipment (Xi’an Zhongke Zhongmei Laser Technology Co., Xi’an, China). The optimum laser cladding process parameters, determined from earlier research, were as follows: laser power of 4500–5000 W, laser diameter of 5 mm, scanning speed of 1.8–3.6 m/s, and powder feed rate of 18 g/min [33]. The parameters for cold spraying and laser cladding are shown in Table 2 and Table 3, respectively.

### 2.3. Performance Test

**Figure 3:**
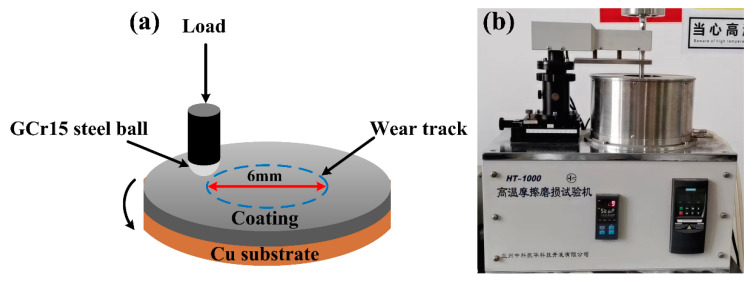
(**a**) Schematic diagram of the tests for friction wear and (**b**) photographs of the test equipment for friction wear.

A Vickers hardness tester (200HBVS-30, Laizhou Huayin Experimental Instrument Co., Laizhou, China) at a load of 5 N for 15 s was used for testing the microhardness of the coatings along the cross-section. The samples were polished before the unlubricated friction wear test. Afterwards, a ball-on-disk Ht-1000 tester (Zhongkekaihua Science and Technology Co., Ltd., Lanzhou, China) with a diameter of 6 mm as the counter-body was used to test the steel balls (GCr15, hardness of 60 HRC) for 30 min, at a load of 20 N, a rotation speed of 400 rpm, and with a corresponding sliding distance of 226 m as shown in Figure 3a,b. The wear rate was calculated using the following equation [12,34,35]:

(1) $$Specific\;wear\;rate\left(m^{3}/Nm\right)=\frac{Wear\;Volume\left(m^{3}\right)}{Normal\;load\left(N\right)\times Sliding\;distance\left(m\right)}$$

The frictional wear track was measured using an ultra-deep field 3D microscope (VHX-5000, Keenes Co., Oban, Japan) and, for each sample, the average wear was calculated three times.

### 2.4. Characterization

Electric discharge machining (EDM) was used to cut all the coating samples to ensure no extra stress was created during the process. Then, the samples were ground and polished using abrasive papers ranging from 400 to 1200 grit and SiC suspension. Then, the samples were etched in an etchant (2.7 g Fe (NO_3_)_3_, 10 mL C_2_H_5_OH, and 10 mL deionized water) for 15 s before being measured using a scanning electron microscopic (SEM, MIRA4 LMH, TESCAN, Czech) that was equipped with energy-dispersive spectroscopy (EDS) to observe the microstructure (cross-sectional) and elemental analysis. To calculate the porosity of the coating, image analysis software employing the SEM micrographs of cross-sectional samples after polishing was used. At the same time, an optical profiler (WYKO NT9100, Veeco Metrology Inc., Plainview, NY, USA) was used to measure the surface roughness of the coating as well as its 3D morphology. In addition, the phase composition of coatings and powders were detected using X-ray diffraction (XRD, Empyren, PANalytical, The Netherlands) employing Cu-Kα radiation of 1.5418 Å with an operating voltage of 45 KV, operating current of 40 mA, 2θ range of 20–80°, and scan rate of 0.02 °/s.

## 3. Results and Discussion

### 3.1. Microstructure of Cold-Sprayed Pre-Set Coating

**Figure 4:**
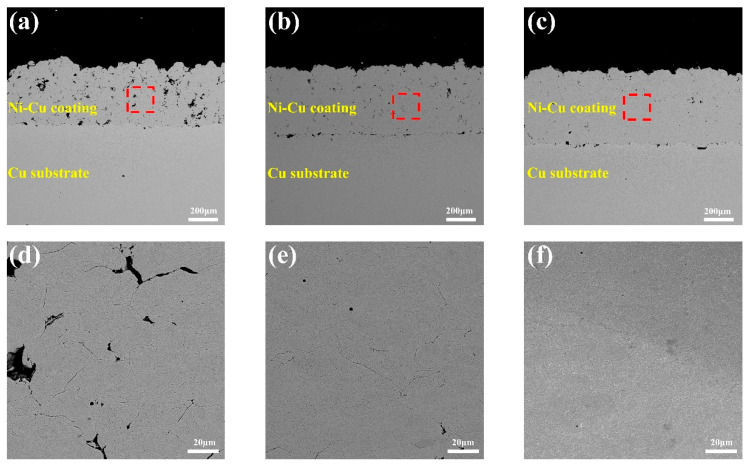
SEM images of coating (**a**–**c**) cross-sectional images of samples ##1, ##2, and ##3, respectively, and (**d**–**f**) magnified internal morphology of the red marked areas of samples ##1, ##2, and ##3, respectively.

The SEM cross-sectional morphologies of cold-sprayed coatings #1, #2, and #3 are shown in Figure 4a–c. The successful deposition of the Ni-Cu alloy coating with a thickness of about 400–600 μm can be observed on the Cu substrate under three different cold spraying processes as seen in Figure 4d–f that showed no obvious defects such as discontinuities and microcracks. From the highly magnified images, the Ni-Cu particles clearly undergo obvious plastic deformation under different processes. The number of micropores of sample ##3 is significantly less than that of sample ##1. This is due to the increasing pressure of the carrier gas and shortening of the spraying distance, which increases the particle velocity to the powder, thus enhancing the compacting and process hardening effects between the particles and consequently improves the deposition rate and quality of the coating [36,37].

**Figure 5:**
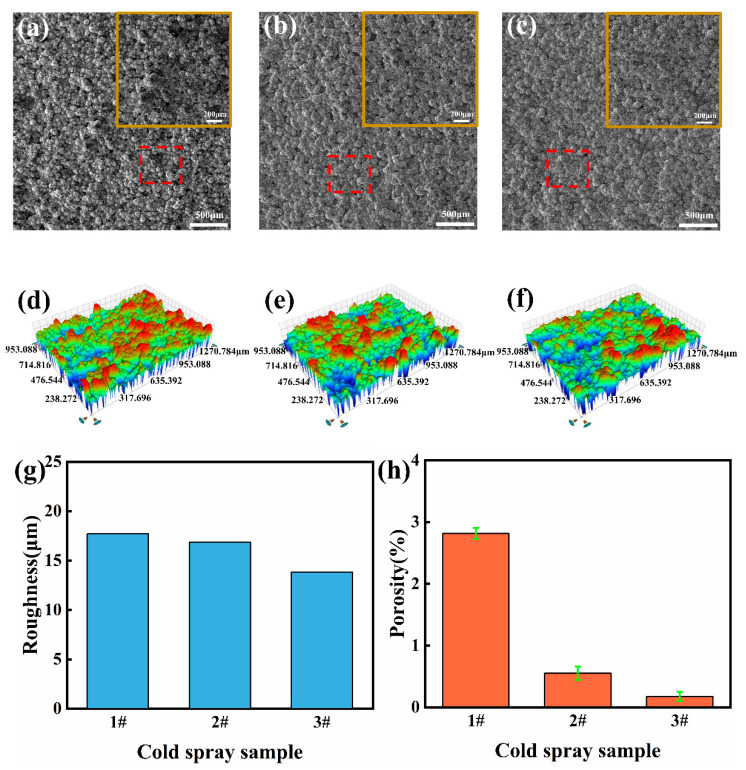
(**a**–**c**) SEM images of coating surface for the samples #1, #2, and #3 (**d**–**f**), respectively; 3D profile of the samples #1, #2, and #3 (**g**), respectively; surface roughness (Ra) and (**h**) porosity of samples #1, #2, and #3.

The SEM images of the surface morphology of cold-sprayed samples ##1, ##2, and ##3 in Figure 5a–c clearly show the spherical shape of the Ni-Cu alloy particles with a large number of pores still visible on the surface of sample ##1. As for samples ##2 and ##3, serious plastic deformation of the Ni-Cu alloy particles from spherical to irregular oval or stretched flat shapes are observable due to the faster particle velocity resulting from the higher carrier gas pressure used that finally causes the coating to be more dense and have a flat surface [38]. As can be seen in the 3D profile of the surface in Figure 5d–f, the surface of sample ##3 is flatter compared to the surface of samples ##1 and ##2.

In addition, as shown in Figure 5g, the surface roughness (Ra) of sample #3 is 13.82 μm, while, for samples #1 and #2, the values are close to 17.7 μm and 16.84 μm, respectively, with both being slightly larger than that of sample #3. More importantly, the porosity of samples #1, #2, and #3, as in Figure 5h, are 2.814%, 0.552%, and 0.176%, respectively This indicates that the porosity of sample #3 is less than 24 times and three times that of sample #1 and #2, respectively. Porosity is known to be one of the important indicators of cold spray coating quality; a lower porosity can improve the coating strength, friction wear, and other properties [39,40]. Based on the surface roughness and porosity values, the samples prepared by cold spraying process #3 were preferred for subsequent laser melting.

### 3.2. Microstructure of Gradient Coating

**Figure 6:**
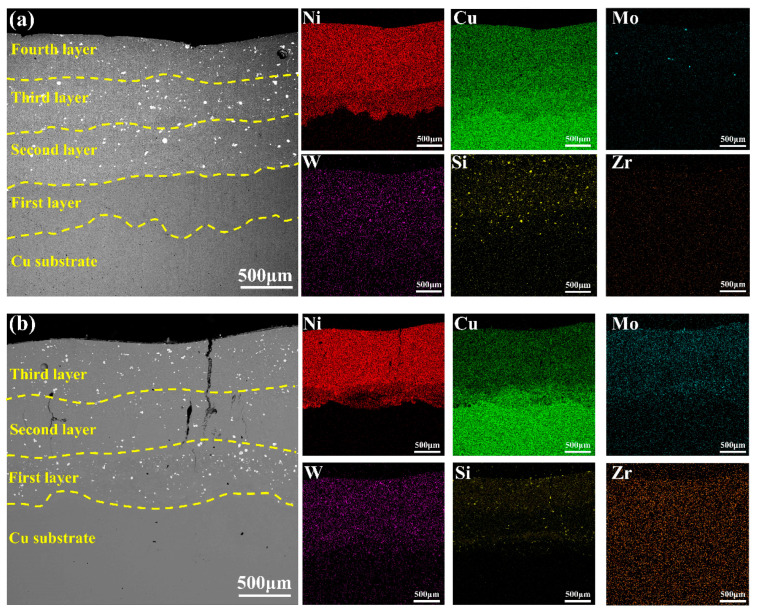
SEM morphologies and the corresponding EDS elemental mapping (wt.%) of Ni, Cu, Mo, W, Si, and Zr of (**a**) cold spray–laser cladding composite gradient coating (CLGC) and (**b**) laser cladding gradient coating (LGC).

The SEM morphologies of the coatings obtained using cold spray–laser cladding composite gradient (CLGC) and laser cladding gradient (LGC) along with their corresponding EDS elemental mapping are shown in Figure 6a,b, respectively. The good quality of CLGC can be observed in Figure 6a, with no defects such as holes and cracks being found. Moreover, from the EDS elemental mapping, it is obvious that elements such as Ni and Cu show gradient distribution inside the coating, with only a small diffusion of the Ni elements into the Cu substrate, thus indicating its small dilution rate and insignificant thermal influence on the Cu substrate. On the other hand, the holes are observed to be generated inside LGC as e seen in Figure 6b, while the cracks are also generated between the interface of the first layer and second layer, which extend to the surface. This is mainly due to the combination of residual thermal stress accumulated during the gradient cladding process and the tensile stress generated by the huge temperature gradient [41,42]. In addition, the EDS elemental mapping shows that a large amount of Cu elements diffused from the substrate into the first layer due to the simultaneous formation of a melt pool on the Cu substrate surface and powder during coaxial laser cladding, thus resulting in a high dilution rate and affecting the performance of the gradient coating.

**Figure 7:**
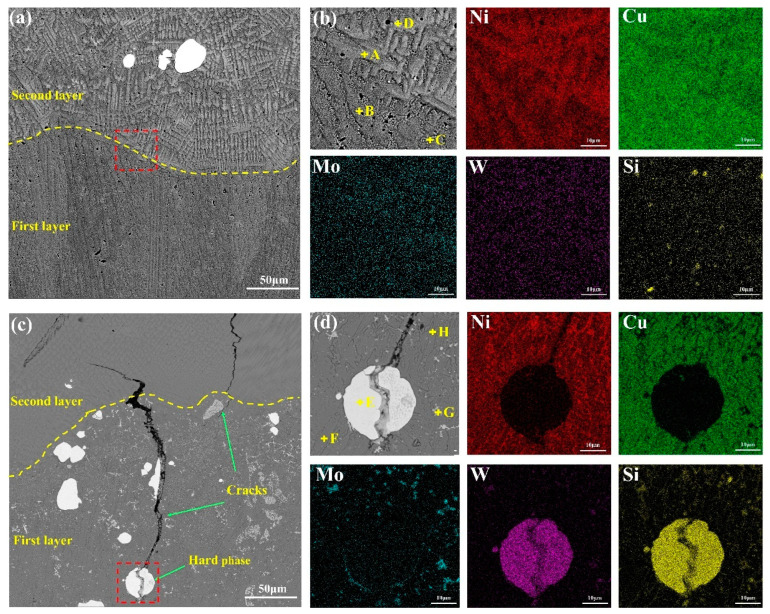
SEM morphologies and the corresponding EDS elemental mapping (wt.%) of: (**a**,**b**) cold spray–laser cladding composite gradient coating (CLGC), (**c**,**d**) laser cladding gradient coating (LGC).

The microstructure and elemental mapping of CLGC and LGC are shown in Figure 7, respectively. Figure 7a,b demonstrates that different layers of CLGC are efficiently bonded to each other without defects such as cracks and holes, but the organization morphology differs significantly. This is mainly due to the large temperature gradient at the interface between the first layer and the Cu substrate generated during rapid solidification. This is conducive to crystallization and growth, resulting in dominant columnar crystals perpendicular to the melt pool in this region, while the second layer is dominated by disordered short rod-like dendrites due to the refinement effect of remelting [43].

In contrast, it is obvious from Figure 7c,d that, for LGC, a number of longitudinal cracks exist between the layers that gradually extend upward. Further, the analysis of the mapping images and the EDS analysis of different points in Table 4 reveal a significant segregation of W, Mo, Si, etc., in the first layer of LGC. As a result, the generation of large number of unevenly distributed hard phases led to the large brittleness and low ductility of LGC; the larger CTE difference between LGC and Cu substrate was because of the lack of a NiCu interlayer also increasing its cracking tendency [42].

### 3.3. XRD Patterns

**Figure 8:**
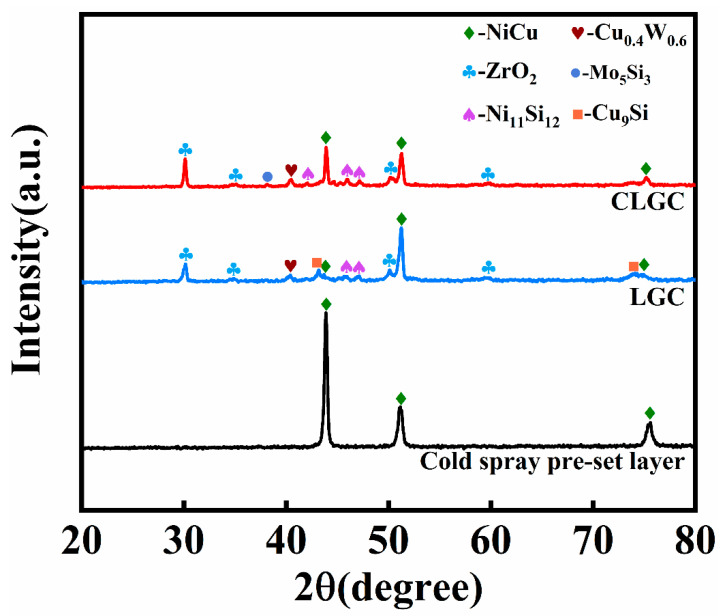
XRD spectra of cold spray–laser cladding composite gradient coating (CLGC), laser cladding gradient coating (LGC), and cold spray pre-set layer.

The XRD curves of CLGC, LGC, and cold spray pre-set layer are shown in Figure 8. It can be directly seen from the figure that, for the cold spray pre-set layer, the diffraction peak only corresponds to NiCu (PDF-65-7246), indicating there was no change of the powder composition during the cold spraying process and no impurities contaminating the first layer. Furthermore, the XRD spectra of CLGC and LGC were almost identical, with the XRD pattern of CLGC showing the appearance of obvious diffraction peaks of ZrO_2_ (PDF-49-1642), Ni_11_Si_12_ (PDF-17-0222), and so on besides the NiCu solid solution phase. This is because, during the process of melt formation, phases such as Ni_11_Si_12_ are formed due to the reaction of various elements among each other. The YSZ particles, on the other hand, have low densities and tend to accumulate on the coating surface thanks to the Marangoni effect. These phases contribute to the tissue refinement of CLGC, which in turn raises its microhardness and improves its wear performance.

### 3.4. Hardness Distribution

**Figure 9:**
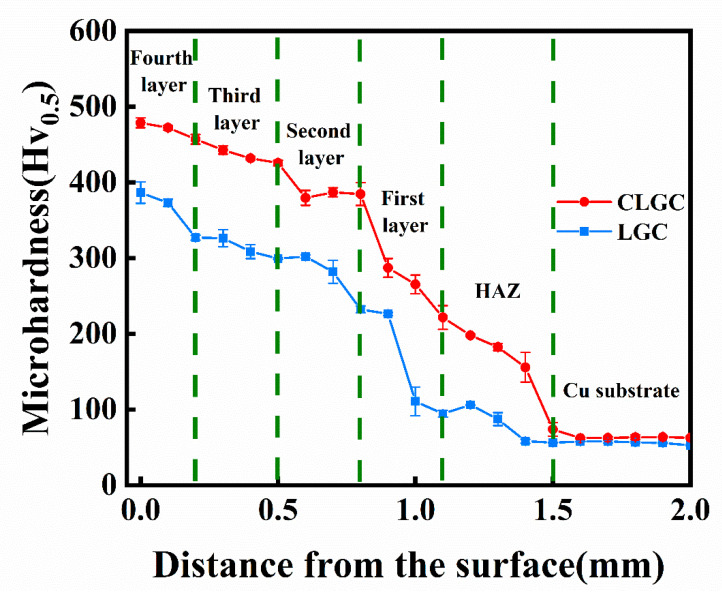
Microhardness of cold spray–laser cladding composite gradient coating (CLGC) and laser cladding gradient coating (LGC).

The curves showing the distribution of microhardness for CLGC and LGC are represented in Figure 9. The overall microhardness of CLGC can be seen to be somewhat higher compared to LGC. This may be attributed to the lack of a Ni-Cu interlayer in LGC and the higher energy input of the laser beam operating directly on the copper substrate increasing the substrate’s diluting effect on the coating and causing a large number of Cu elements to enter the coating, thus influencing the overall microhardness of LGC. On the other hand, the coating’s density lowering due to the existence of defects such as cracks and pores inside LGC as seen in Figure 6b also impact its microhardness [8,44]. It is worth noting that the value of the average microhardness of the fourth layer of CLGC is 478.8 HV_0.5_, being almost eight times that of the Cu substrate. This increasing microhardness is due to the formation of hard intermetallic compounds such as Ni_11_Si_12_ and Mo_5_Si_3_ and the strengthening effect of the Ni-Cu solid solution [33]. More importantly, in addition to reflecting the gradient change of its coating composition, the gradient distribution of the CLGC microhardness also improves the overall anti-wear properties and bond strength between the two neighboring layers of the coating [12].

### 3.5. Friction and Wear Performance

**Figure 10:**
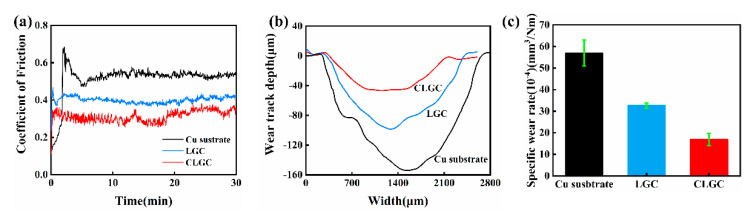
(**a**) Friction coefficient, (**b**) Wear track cross-section profiles, and (**c**) Specific wear rate of cold spray-laser cladding composite gradient coating (CLGC) and laser cladding gradient coating (LGC).

The friction coefficient, wear volume, and wear morphology are the terms for evaluating the frictional property, which is a very important macroscopic characteristic of laser clad coatings. The curves corresponding to the coefficient of friction (CoF) of CLGC, LGC, and Cu substrate tested for 30 min are shown in Figure 10a. Generally, smaller values of CoF represent better anti-wear properties. The figure shows that the respective values of CoF for CLGC and LGC are 0.27 and 0.43 and that both are significantly lower than the Cu substrate for which the value is 0.54. This is due to the improvement of the microhardness of the coating that results from the formation of hard phases such as Mo_5_Si_3_ during the laser cladding process and the wear resistance behavior of the YSZ particles that, together, improve the wear resistance of CLGC [9,41]. On the other hand, the slightly lower CoF of CLGC compared to that of LGC may be because of the large numbers of pores and cracks inside LGC, as shown in Figure 6b, which cause the wear debris and increase the contact area during sliding, thus leading to an increase in the friction coefficient.

To further investigate the wear resistance, the cross-sectional profiles and specific wear rates after the wear testing of the coating and Cu substrate are shown in Figure 10b,c, respectively. The wear track of CLGC is visibly quite shallow and narrow compared to LGC and Cu substrate, as evident in Figure 10b. In addition, the respective specific wear rate values were calculated and are provided in Figure 10c (16.89 × 10^−4^ mm^3^/Nm (CLGC), 32.64 × 10^−4^ mm^3^/Nm (LGC), and 56.97 × 10^−4^ mm^3^/Nm (Cu substrate)), which clearly shows that high hardness, good bonding, and the absence of cracks and holes in CLGC greatly enhance the wear resistance of the Cu substrate.

**Figure 11:**
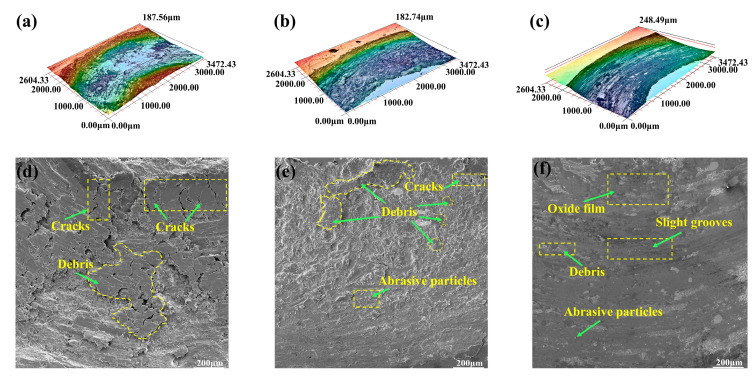
The 3D wear and SEM morphologies of (**a**,**d**) Cu substrate, (**b**,**e**) laser cladding gradient coating (LGC), and (**c**,**f**) cold spray–laser cladding composite gradient coating (CLGC).

Figure 11 shows the 3D and SEM wear morphology of each sample. Comparing the 3D morphology of Figure 11a–c under the same test conditions, the wear width of the Cu substrate is 2339.06 μm with the presence of large debris on the surface, whereas the wear width of CLGC is only 1820.13 μm with slight grooves on the wear surface, indicating the better wear resistance of CLGC. Furthermore, Figure 11d clearly shows the presence of a large amount of wear debris and a few cracks on the surface of the Cu substrate. This is due to the surface of Cu being prone to continuous plastic deformation under contact stress because of the low hardness, thus resulting in large area tearing, which is a typical adhesive wear [45]. In addition, Figure 11e demonstrates that, after the friction wear test, the surface of LGC is relatively flat and, though it is a great improvement over the Cu substrate, the peeling of the coating material still results in some wear debris, cracks, and pits.

In contrast, Figure 11f shows the relatively smooth surface of CLGC with only slight grooves and few wear particles, thus indicating that abrasive wear is its wear mechanism. Compared with LGC and the Cu substrates, the hard phases such as Ni_11_Si_12_ and Mo_5_Si_3_ generated in the cladding of CLGC can effectively resist plastic shear, reduce the tearing phenomenon, and be attributed to the improved wear resistance of CLGC. On the other hand, non-uniform and non-equilibrium rapid solidification organization and the lack of defects such as cracks and holes result in good strength and toughness of CLGC, thus providing it with excellent resistance to spalling and delamination [46,47]. In conclusion, CLGC has good frictional wear properties.

**Figure 12:**
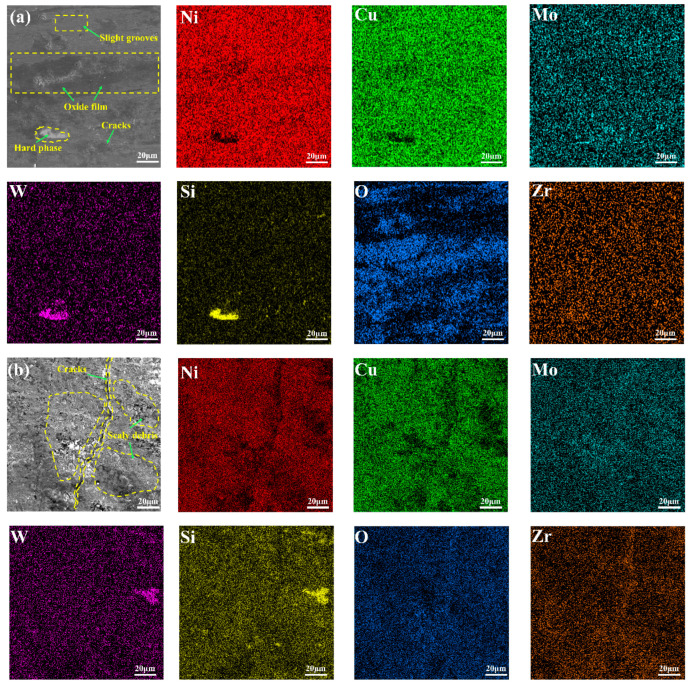
SEM morphologies showing the worn surface and the corresponding EDS elemental mapping (wt.%) of (**a**) cold spray–laser cladding composite gradient coating (CLGC) and (**b**) laser cladding gradient coating (LGC).

For further investigating the wear mechanism, the SEM surface wear morphology of CLGC and LGC and their corresponding elemental distributions are shown in Figure 12. Figure 12a shows the wear morphology of CLGC in which its surface is relatively flat, it has no obvious abrasive debris or pits, and there are only a few slight grooves, thus indicating that there is abrasive wear during the wear process of CLGC. At the same time, the presence of a large amount of O in the wear surface is evident in its mapping; the rapid oxidation of the surface due to the high-contact friction temperature generated during the wear process may be responsible for the oxide film. The dense oxide film can improve the wear resistance of CLGC by withstanding the positive stress and separating the opposite wear surface [48].

On the other hand, for LGC, the surface is heavily worn and the peeling of the coating material as well as shallow and large scaly debris can be observed close to the cracked area as evident in Figure 12b. In the wear process, the crack sprouting and expansion of LGC can be attributed to the fact that, when the pairs of grinding balls and the LGC surface contact each other, defects such as pores on the surface layer of LGC become the source of stress concentration; thus, cracks eventually form when the contact stress exceeds the fatigue strength of the material and continuously extend and expand to the inside, bifurcating into shedding pits after a certain depth [49]. All these indicate the occurrence of surface fatigue wear in LGC during the wear process.

**Figure 13:**
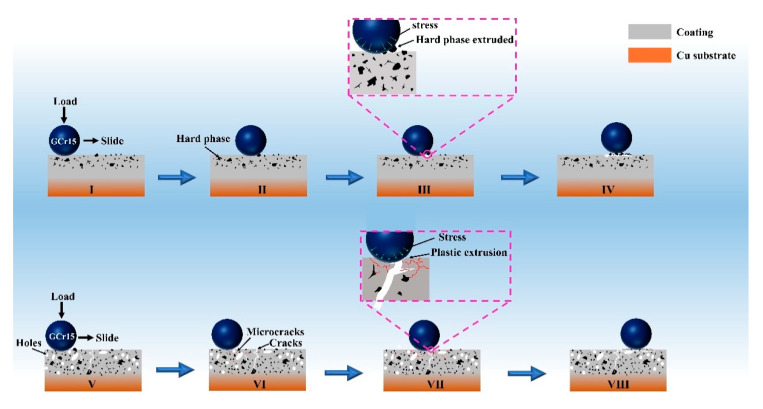
Schematic illustration of the wear mechanism of (I–IV) cold spray–laser cladding composite gradient coating (CLGC) and (V–VIII) laser cladding gradient coating (LGC).

Figure 13 (I–IV) and (V–VIII) are schematic diagrams of the possible wear mechanisms of CLGC and LGC, respectively. In this case, the stresses on the coating from the GCr15 steel balls are simplified to compressive stresses acting in the normal direction and shear stresses acting in the sliding direction (Illustration I). During the frictional wear of CLGC, hard particles, which are present in large quantities on the CLGC surface, come into contact with each other and are separated from the CLGC by the frictional stress, leaving craters on the wear surface (Illustrations II–III). When detachment produces more hard particles on the coating surface, they accumulate in the area of mutual contact on the wear surface and form three-body abrasive wear, resulting in plow grooves and plastic deformation on the CLGC surface (Illustration IV).

In the frictional wear process of LGC, due to the presence of a large number of defects such as holes and cracks within LGC, it causes these locations to be prone to stress concentration under load stress, resulting in fatigue wear and, when the wear accumulates to a certain extent, fatigue microcracks being generated at the defect locations (Illustration V–VI). As the wear process intensifies, these fatigued microcracks expand to the inner surface of the coating and connect with the original cracks. At the same time, under stress, when cracks in different directions connect with each other, the surface coating is plasticly extruded, peeling off and forming spalling pits (Illustration VII). The debris peeling off from the coating surface is involved in the further wear process (Illustration VIII).

## 4. Conclusions

Nickel-based gradient coatings were successfully prepared on Cu substrates using a composite method of cold spraying and laser cladding. The microstructure, hardness, and wear properties of the coatings were investigated. The following conclusions can be drawn:

(1) The combined method of cold spraying and laser remelting effectively reduces the difference in the coefficients of thermal expansion (CTE) between the Ni-Cu interlayer coating and the Cu substrate, thus reducing the tendency of the coating to crack.

(2) The transition between each layer composition for the gradient coating is uniform without obvious abrupt changes, while the diffusely distributed hard phases, such as Ni_11_Si_12_ and Mo_5_Si_3_, improve the microhardness of the coating.

(3) The microhardness of the gradient coating (478.8 HV_0.5_) is almost eight times that of the Cu substrate (62.1 HV_0.5_) and the wear rate is only one-third that of the substrate, thus providing excellent wear resistance. The wear mechanisms are abrasive wear and oxidation wear.

## Figures and Tables

**Table 1:** Chemical composition of feedstock powder and substrate.

Material	Chemical Composition (wt.%)										
	Ni	Cu	Mo	W	Si	As	Fe	Sn	S	YSZ	Others
Fourth layer	40.00	30.00	7.32	14.04	8.64					1	
Third layer	50.00	30.00	4.88	9.36	5.76						
Second layer	60.00	30.00	2.44	4.68	2.88						
First layer	70.00	30.00									
substrate		99.9				0.002	0.008	0.008	0.005		<0.1

**Table 2:** The conditions used in the cold spraying process.

Samples	Gas Pressure (Mpa)	Gas Temperature (°C)	Spray Distance (mm)	Accelerating Gas	Spray Angle (°)
#1	3	800	40	N_2_	90
#2	4	700	20	N_2_	90
#3	5	800	20	N_2_	90

**Table 3:** The conditions used in the laser cladding process.

Coating	Laser Power (w)	Laser Beam Diameter (mm)	Scanning Speed (m/min)	Powder Feeding Rate (g/min)
First layer	5000	5	1.8	
Second layer	4500	5	1.8	18
Third layer	5000	5	3.6	18
Fourth layer	5000	5	3.6	18

**Table 4:** EDS point analysis results of each point marked in Figure 7 (wt.%).

Point	Ni	Cu	Mo	W	Si
A	32.08	42.37	8.16	17.39	0.00
B	18.17	81.04	0.00	0.29	0.50
C	18.08	81.82	0.00	0.00	0.10
D	18.53	48.50	0.38	0.07	32.52
E	0.60	0.88	0.00	98.46	0.06
F	23.49	73.89	0.18	0.64	1.79
G	15.40	12.72	31.67	38.96	1.26
H	52.58	36.50	0.77	0.44	9.71

## Data Availability

Not applicable.
